# Regulation of tumour related genes by dynamic epigenetic alteration at enhancer regions in gastric epithelial cells infected by Epstein-Barr virus

**DOI:** 10.1038/s41598-017-08370-7

**Published:** 2017-08-11

**Authors:** Atsushi Okabe, Sayaka Funata, Keisuke Matsusaka, Hiroe Namba, Masaki Fukuyo, Bahityar Rahmutulla, Motohiko Oshima, Atsushi Iwama, Masashi Fukayama, Atsushi Kaneda

**Affiliations:** 1 Department of Molecular Oncology, Graduate School of Medicine, Chiba University, Chiba, Japan; 2 Department of Cellular and Molecular Medicine, Graduate School of Medicine, Chiba University, Chiba, Japan; 3 Department of Pathology, Graduate School of Medicine, The University of Tokyo, Tokyo, Japan

## Abstract

Epstein-Barr virus (EBV) infection is associated with tumours such as Burkitt lymphoma, nasopharyngeal carcinoma, and gastric cancer. We previously showed that EBV(+) gastric cancer presents an extremely high-methylation epigenotype and this aberrant DNA methylation causes silencing of multiple tumour suppressor genes. However, the mechanisms that drive EBV infection-mediated tumorigenesis, including other epigenomic alteration, remain unclear. We analysed epigenetic alterations induced by EBV infection especially at enhancer regions, to elucidate their contribution to tumorigenesis. We performed ChIP sequencing on H3K4me3, H3K4me1, H3K27ac, H3K27me3, and H3K9me3 in gastric epithelial cells infected or not with EBV. We showed that repressive marks were redistributed after EBV infection, resulting in aberrant enhancer activation and repression. Enhancer dysfunction led to the activation of pathways related to cancer hallmarks (e.g., resisting cell death, disrupting cellular energetics, inducing invasion, evading growth suppressors, sustaining proliferative signalling, angiogenesis, and tumour-promoting inflammation) and inactivation of tumour suppressive pathways. Deregulation of cancer-related genes in EBV-infected gastric epithelial cells was also observed in clinical EBV(+) gastric cancer specimens. Our analysis showed that epigenetic alteration associated with EBV-infection may contribute to tumorigenesis through enhancer activation and repression.

## Introduction

Aberrant DNA methylation is one of the major epigenomic alterations that affect cancer development^1–3^. Epstein-Barr virus (EBV) infection is associated with tumours such as Burkitt lymphoma, nasopharyngeal carcinoma (NPC), and some gastric cancer. Among all human malignancies, NPC and EBV(+) gastric cancer (GC) presents an extremely high-methylation epigenotype. This aberrant DNA methylation causes silencing of multiple tumour suppressor genes (TSGs)^4–6^.

Other than DNA methylation, global alteration of heterochromatin-associated histone modifications, H3K9me2/3 and H3K27me3, were observed in many cancer cells. The Polycomb Repressive Complex 2 (PRC2) was mutated in many cancers such as lymphoma or malignant peripheral nerve sheath tumours and, consequently, elevation or reduction of H3K27me3 was observed^7^. H3K27me3 deregulation leads to differentiation and aberrant activation of cancer-associated genes. McDonald *et al*. reported that large H3K9-modified heterochromatin domains were changed during the progression of pancreatic ductal adenocarcinoma, and this change was related to metastasis^8^. In NPC, subunits of PRC2, *BIM1* and *EZH2*, are highly activated^9, 10^, and consequent increase of H3K27me3 is significantly associated with patients’ poor survival and chemotherapy resistance^11^. We previously showed that *SUZ12* and *BMI1* are upregulated also in EBV-infected gastric epithelial cells^12^, which might cause repressive mark alteration.

In addition to heterochromatin regions, enhancers are also important regulators of tissue-specific expression, and deregulation of enhancers by mutation or epigenetic alteration could lead to diseases^13^. Mutations in enhancers and enhancer-binding transcription factors or mutations in cofactors cause hyper-activation or repression of enhancers that provoke cancer. It was also reported that *MLL3* and *MLL4* genes, which are methyl-transferase of enhancer mark H3K4me1, were frequently mutated in many digestive cancers, including colon cancer or GCs^14–16^. Hyper-activated enhancer cluster, which is called super enhancer, defines cell identity in health and disease, and was analysed in many cancer cells^17, 18^. It was reported that GC-related somatic super enhancers contribute to cancer gene expression^19^.

EBV infection could also induce epigenetic alteration and concomitant deregulation of expression, which have been mostly studied by using permanently growing EBV-infected lymphoblastoid cell lines (LCLs)^20–25^. Several previous reports showed DNA methylation induction and aberrant expression alteration in EBV-infected epithelial cells^26, 27^. Ryan and colleagues reported 17 downregulated and eight upregulated genes by EBV using the gastric cancer cell line AGS, with or without EBV infection^28^. However, epigenetic alterations, other than DNA methylation, during tumorigenic processes after EBV infection are yet to be fully investigated.

In this study, we analysed alterations of histone modification to evaluate how EBV infection plays a role in viral-associated carcinogenesis. We used a system *in vitro* to infect a gastric epithelial cell line with EBV which can induce an extensive DNA hypermethylation^5^. We investigated that, while loss of repressive marks at promoter regions did not lead to gene activation due to alternatively acquired *de novo* DNA methylation, loss of repressive marks at enhancer regions resulted in enhancer activation due to alternatively acquired histone active marks. Activation and repression of enhancer regions in EBV-infected GC cells leading to aberrant activation of oncogenes and repression of TSGs, and these genes were preferentially deregulated by epigenetic alteration at enhancer regions rather than at promoter regions. This comprehensive analysis of epigenetic alteration in EBV-infected GC cells provides insights into the molecular mechanisms underlying the development of EBV(+) GC.

## Results

### Epigenetic alteration at promoters and enhancers in EBV-infected GC cells

To identify genome-wide epigenetic alterations in EBV-infected GC cells, we performed ChIP-seq analyses for H3K4me3, H3K4me1, H3K27ac, H3K27me3, and H3K9me3 using a low-methylation GC cell line, MKN7, and one of the previously established EBV-infected MKN7 clones^5^. To clarify the relationship between *de novo* DNA methylation and each histone modification alterations, we performed ChromHMM analysis using ChIP-seq data and divided the genome into 9 chromatin states (Supplementary Fig. S1a). We then compared each histone modification alteration with DNA methylation alteration in EBV-infected MKN7 cells. Consistent with previous results, *de novo* DNA methylation was preferentially acquired at repressive and poised promoter regions. In addition, active enhancer, polycomb target regions, and heterochromatin regions also acquired DNA methylation (Supplementary Fig. S1b). These results suggest that EBV infection could induce *de novo* DNA methylation on promoter regions as well as on enhancers and repressive regions.

**Figure 1:**
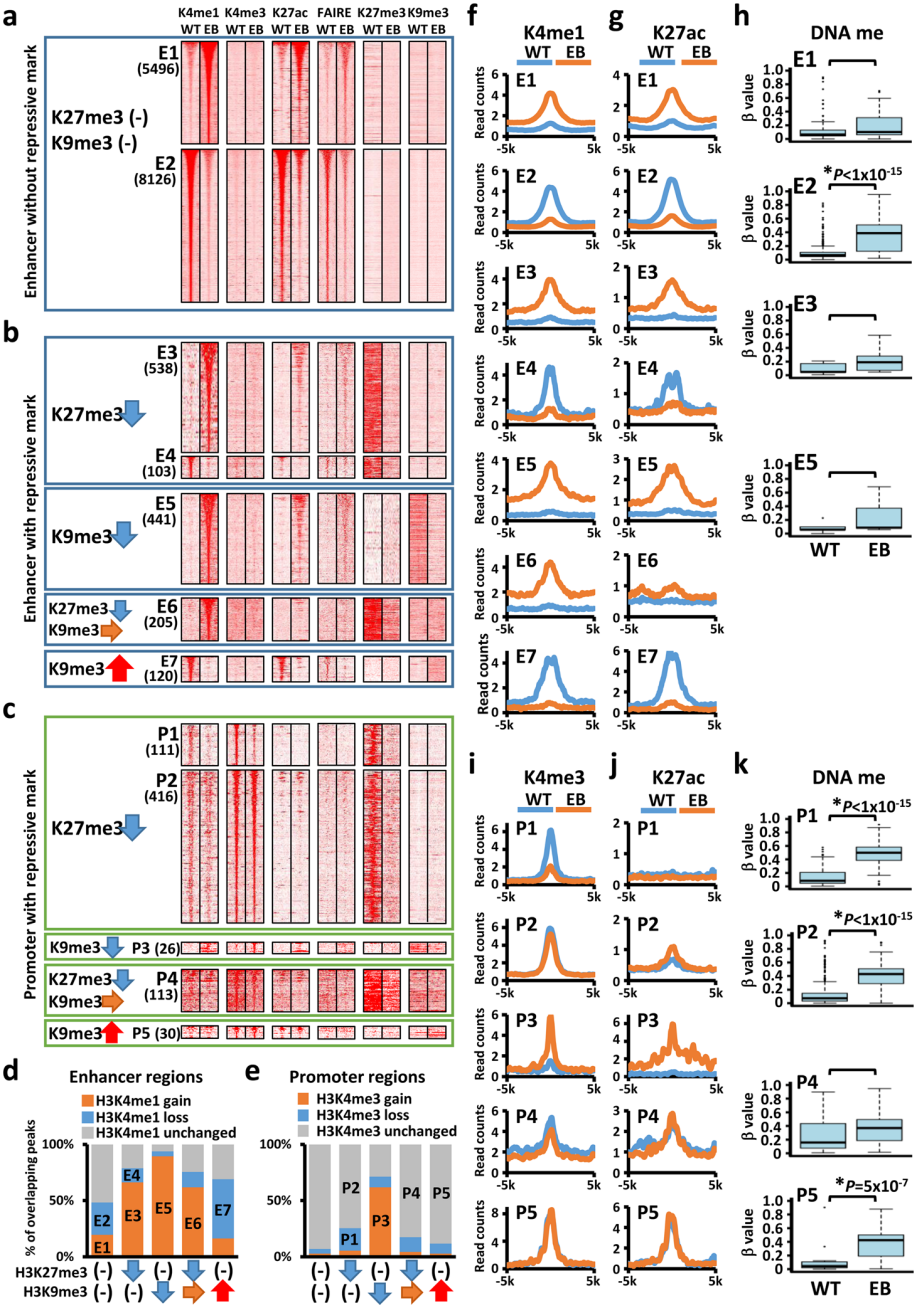
Global epigenetic alteration at enhancer and promoter regions. *WT*, MKN7 without EBV infection. *EB*, EBV-infected MKN7 cells. (**a**,**b**) Histone modification alteration at enhancer regions with or without repressive marks. Heatmaps represent read density of H3K4me1, H3K4me3, H3K27ac, FAIRE, H3K27me3, and H3K9me3 profiles in ± 5-kb regions centred on H3K4me1 peaks without H3K27me3 and H3K9me3 modifications (**a**), or with H3K27me3 or H3K9me3 alteration (**b**). H3K4me1 level was increased in subgroups E1, E3, E5, and E6, while H3K4me1 level was decreased in subgroups E2, E4, and E7. (**c**) Histone modification alteration at promoter regions with repressive marks. Read densities of H3K4me1, H3K4me3, H3K27ac, FAIRE, H3K27me3, and H3K9me3 in ± 5-kb regions are represented in heatmaps, centred on H3K4me3 peaks. H3K4me3 level was decreased in P1, and increased in P3, while H3K4me3 level was not changed in P2, P4, and P5. (**d**) Fractions of each enhancer subgroup in H3K4me1 gain, loss, or unchanged peaks. (**e**) Fractions of each promoter subgroup in H3K4me3 gain, loss, or unchanged regions. (**f**) Profiles of average H3K4me1 distribution at each enhancer subgroup. (**g**) Profiles of average H3K27ac distribution at each enhancer subgroup. (**h**) Average DNA methylation level at the centre of each enhancer. *Significant increase (*P* < 0.05: paired *t*-test) of β value from < 0.2 to > 0.4. (**i**) Profiles of average H3K4me3 distribution at each promoter subgroup. (**j**) Profiles of average H3K27ac distribution at each promoter subgroup. (**k**) Average DNA methylation level at overlapping gene promoters. * Significant increase (*P* < 0.05: paired *t*-test) of β value from < 0.2 to > 0.4.

To investigate the effect of *de novo* DNA methylation on enhancer regions, we analysed H3K4me1 alteration on a genome-wide scale after EBV infection (Fig. 1). We defined enhancer regions as H3K4me1 peaks without H3K4me3 signal. First, we identified 5,496 enhancers with increased H3K4me1 signal (E1) and 8,126 enhancers with decreased H3K4me1 signal (E2) (Fig. 1a,f). Enhancer subgroup E1 showed increase of H3K27ac level (Fig. 1g) with a DNA methylation level <0.2 (Fig. 1h), whereas subgroup E2 showed decrease of H3K27ac level (Fig. 1g) with a DNA methylation level significantly increased to >0.4 (*P* < 1 × 10^−15^, paired *t*-test) (Fig. 1h). Enhancers without repressive marks were preferentially repressed by *de novo* DNA methylation and concomitant decrease of H3K27ac.

Next, we explored enhancer regions associated with alteration of repressive histone modification levels in EBV-infected MKN7 cells, e.g. H3K27me3 and H3K9me3. According to ChromHMM chromatin states, occupancy of polycomb target regions was notably reduced in EBV-infected MKN7 cells (Supplementary Fig. S2a). These H3K27me3 reductions were observed at promoters or gene body regions (Supplementary Fig. S2b). The average distribution of H3K27me3 signal on genetic regions revealed that the H3K27me3 signal at promoter regions was significantly reduced (Supplementary Fig. S2c). Besides, genome-wide analysis revealed that H3K9me3 distribution was widely changed. Alteration of H3K9me3 signal was inversely correlated with alteration of activation marks (Supplementary Fig. S3a). H3K9me3 level was increased at 9% of H3K9me3 enriched regions and decreased at 22% of H3K9me3 enriched regions (Supplementary Fig. S3b). At enhancer regions showing H3K4me1 peaks before and/or after EBV infection, 65% of enhancers with decreased H3K27me3 showed increase of H3K4me1 levels (E3) and only 13% showed decrease of H3K4me1 levels (E4) (Fig. 1b,d,f). H3K27ac signal in the E3 subgroup was also preferentially increased (Fig. 1g) and DNA methylation level was maintained at <0.2 (Fig. 1h). Epigenetic switch from repressive H3K27me3 mark to active marks could occur at enhancer regions. Besides, 88% of enhancer regions with decreased H3K9me3 showed an increase of H3K4me1 levels (E5) and only 1% showed loss of H3K4me1 peaks (Fig. 1b,d,F). H3K27ac signal in the E5 subgroup was also preferentially increased (Fig. 1g) and DNA methylation level was not significantly altered. These results showed that switch from repressive H3K9me3 mark to active marks could also occur at enhancer regions. Interestingly, H3K4me1 level was increased, but H3K27ac level was not increased at enhancer regions with loss of H3K27me3 and maintenance of H3K9me3. Furthermore, 53% of enhancers with increased H3K9me3 showed decrease of H3K4me1 (E7), which concomitantly reduced H3K27ac signal (Fig. 1b,f,g). These results suggest that enhancers with maintained or increasing H3K9me3 levels were preferentially repressed without H3K27ac induction.

Finally, we identified alterations at H3K4me3(+) promoter regions with repressive marks. A percentage of 19.9% of promoter regions losing H3K27me3 showed decreased H3K4me3 level (P1) and 74.5% showed unchanged H3K4me3 level (P2) (Fig. 1c,e,i). Consistent with previous results, DNA methylation was significantly increased at P1 and P2 promoter regions (*P* < 1 × 10^−15^, paired *t*-test) (Fig. 1k)^29^﻿. H3K27me3(+) promoters preferentially acquired *de novo* DNA methylation and were hardly activated in EBV-infected MKN7 cells, despite enhancer activation associated with decrease of H3K27me3 (Fig. 1g). On the other hand, 62% of promoters with decreased H3K9me3 (P3) showed simultaneous increase of H3K4me3 and H3K27ac levels (Fig. 1e,i,j). These results suggest that H3K9me3 reduction might contribute to promoter activation as observed at enhancer regions. Most P4 and P5 promoter subgroups, which maintained or gained H3K9me3 modification, did not show increase of H3K4me3 levels (Fig. 1i). These subgroups showed high H3K27ac signal (Fig. 1j), but increase of DNA methylation (Fig. 1k).

### Epigenetic status of EBV genome in EBV infected GC cells

We identified epigenetic status of EBV genome in EBV-infected MKN7 cells by using ChIP-seq data. Whereas most parts of EBV genome were modified with repressive marks e.g. H3K9me3 or H3K27me3 (Supplementary Fig. S4a), there were several regions activated with H3K27ac. One of those was observes at Q promoter of EBNA1 (Qp-EBNA1) (Supplementary Fig. S4b). Activation with H3K27ac was also observed at EBER1, EBER2 and OriP, which are reportedly activated by EBNA1 in latent state of EBV in LCLs^30^. Although H3K27ac signal was rather weak, strong FAIRE signal was identified at the promoter region of LMP2A, which is also active in latent state of EBV.

### Genes activated with increase of H3K27ac mark at promoter and enhancer regions

**Figure 2:**
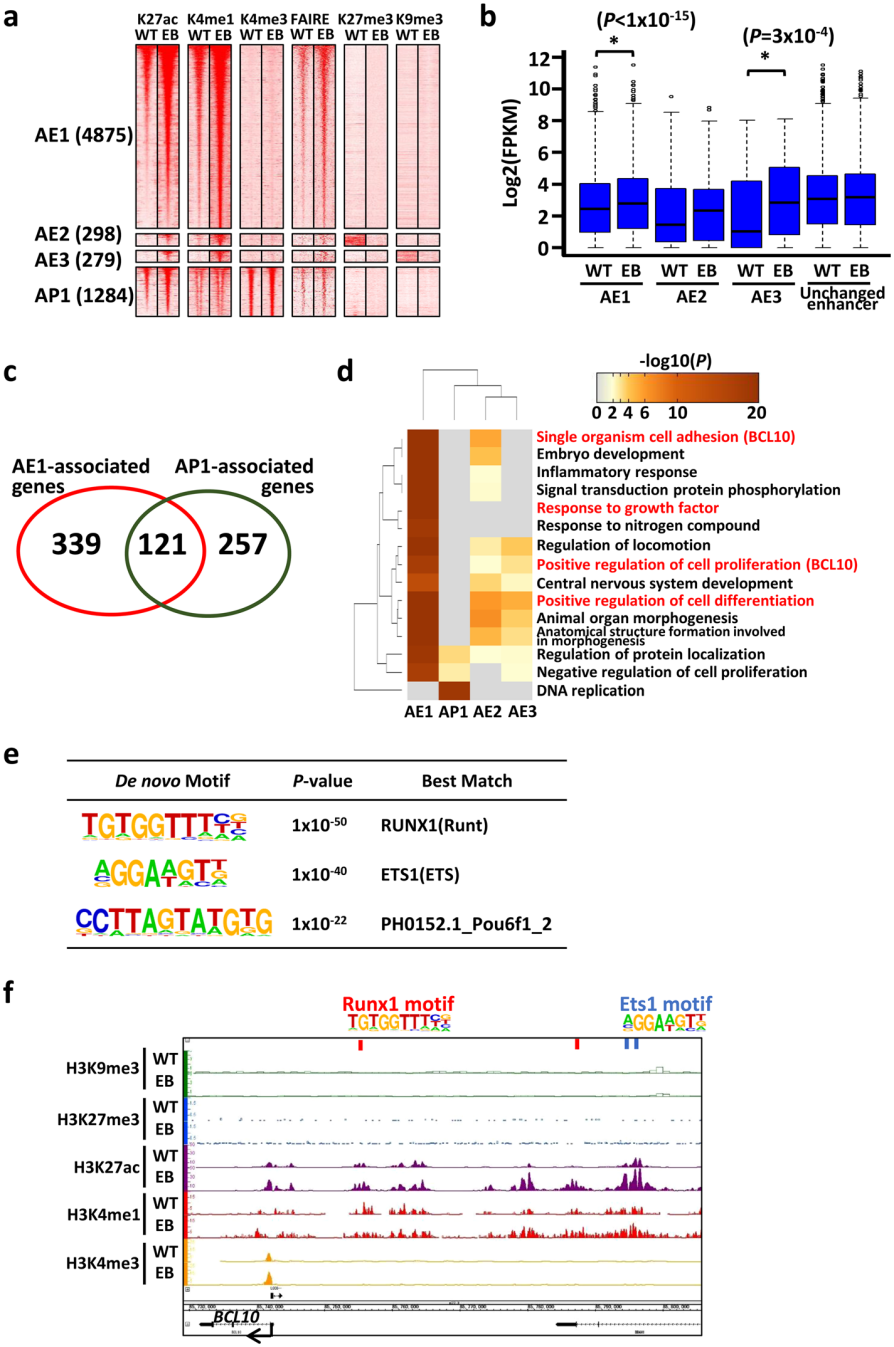
Epigenetic activation of enhancers and promoters in EBV-infected MKN7 cells. *WT*, MKN7 without EBV infection. *EB*, EBV-infected MKN7 cells. (**a**) Heatmaps representing read densities of H3K27ac, H3K4me1, H3K4me3, FAIRE, H3K27me3, and H3K9me3 in ± 5-kb regions centred on activated enhancers and promoters. AE1, AE2, and AE3 are subgroups of activated enhancers in E1, E3, and E5, respectively. AP1 is subgroup of activated promoters without repressive histone modification. (**b**) Expression levels of genes nearest to each activated enhancer and H3K27ac unchanged enhancers. Genes were significantly upregulated in EBV-infected GC cells in AE1 (*P* < 1 × 10^−15^: calculated by paired *t*-test) and AE3 (*P* = 3 × 10^−4^: calculated by paired t-test). (**c**) Overlap of AE1-associated genes (*red*) and AP1-associated genes (*green*). This Venn diagram showed that as many as 339 genes were upregulated by enhancer activation without increase of H3K27ac at promoter. (**d**) GO enrichment analysis of genes with enhancer or promoter activation. Colour scale represents binomial *P*-values (in −log_10_ scale). While genes associated with “DNA replication” are preferentially regulated by promoter activation, those associated with “cell adhesion”, “proliferation”, and “differentiation” are preferentially regulated by enhancer activation. (**e**) Enriched known motif at open chromatin region detected by FAIRE-seq overlapped with AE1. (**f**) Histone modification and position of enriched motifs around the representative activated gene, *BCL10*.

We sought to determine whether a subset of enhancers could be abnormally activated in EBV-infected MKN7 cells. We defined activation as >2-fold H3K27ac signal alterations after EBV infection. We identified 4,875 activated enhancers without repressive marks (AE1), 298 activated enhancers with decrease of H3K27me3 (AE2), 279 activated enhancers with decrease of H3K9me3 (AE3), and 1,284 activated promoters without repressive marks (AP1) (Fig. 2a). EBV infection activates *MAX* enhancers, which were reported as the EBV specific super-enhancer in LCLs^20^ (Supplementary Fig. S5a). Another well-known target of EBNA proteins, *MYC*, was already activated in MKN7 GC cells before EBV infection (Supplementary Fig. S5b). To clarify the contribution of these activated enhancers toward gene expression, we analysed the expression of the nearest gene of each activated enhancer region. Genes associated with AE1 and AE3 showed significantly higher expressions in EBV-infected cells than in WT (*P* < 1 × 10^−15^ for AE1; *P* = 0.0003 for AE3, paired *t*-test) (Fig. 2b). The expression of genes associated with AE2 was not significantly increased, but it tended to be upregulated. Whereas there was an overlap of AE1-associated genes and AP1-associated genes (Fig. 2c), as many as 339 genes were upregulated by enhancer activation without increase of H3K27ac at promoters (Fig. 2c). Therefore, enhancer activation is suggested to be important for aberrant gene activation in EBV-infected GC cells.

Next, we performed GO enrichment analysis of each enhancer subgroup-associated genes to identify activated pathways in EBV-infected MKN7 cells. This analysis revealed significant enrichment of the following categories: regulation of cell migration, single organismal cell-cell adhesion, and response to growth factors for AE1-associated genes (Supplementary Table S1). AE2-associated genes showed enrichment of the following categories: response to external stimulus, differentiation and cell adhesion (Supplementary Table S2). AE3-associated genes showed enrichment of the following categories: cell development and morphogenesis (Supplementary Table S3). Consistent with a previous study, AP1-associated genes showed enrichment of the following categories: DNA replication and cell cycle (Supplementary Table S4)^29^. Clustering analysis of GO enrichment terms revealed that, while genes associated with DNA replication are preferentially regulated by promoter activation, those associated with cell adhesion, proliferation, and differentiation are preferentially regulated by enhancer activation (Fig. 2d).

To predict transcription factors that activate enhancer regions in EBV-infected MKN7 cells, we performed motif analysis at open chromatin regions detected by FAIRE-seq overlapped with AE1. Consensus sequence of RUNX1 and ETS1 were highly enriched in AE1 regions (Fig. 2e). For example, activated enhancers were observed around *BCL10*, which was included in the following GO categories: single organism cell adhesion and positive regulation of cell proliferation; RUNX1 and ETS1 motifs were also observed at activated enhancer regions around this gene.

### Genes repressed with decrease of H3K27ac mark at promoter and enhancer regions

**Figure 3:**
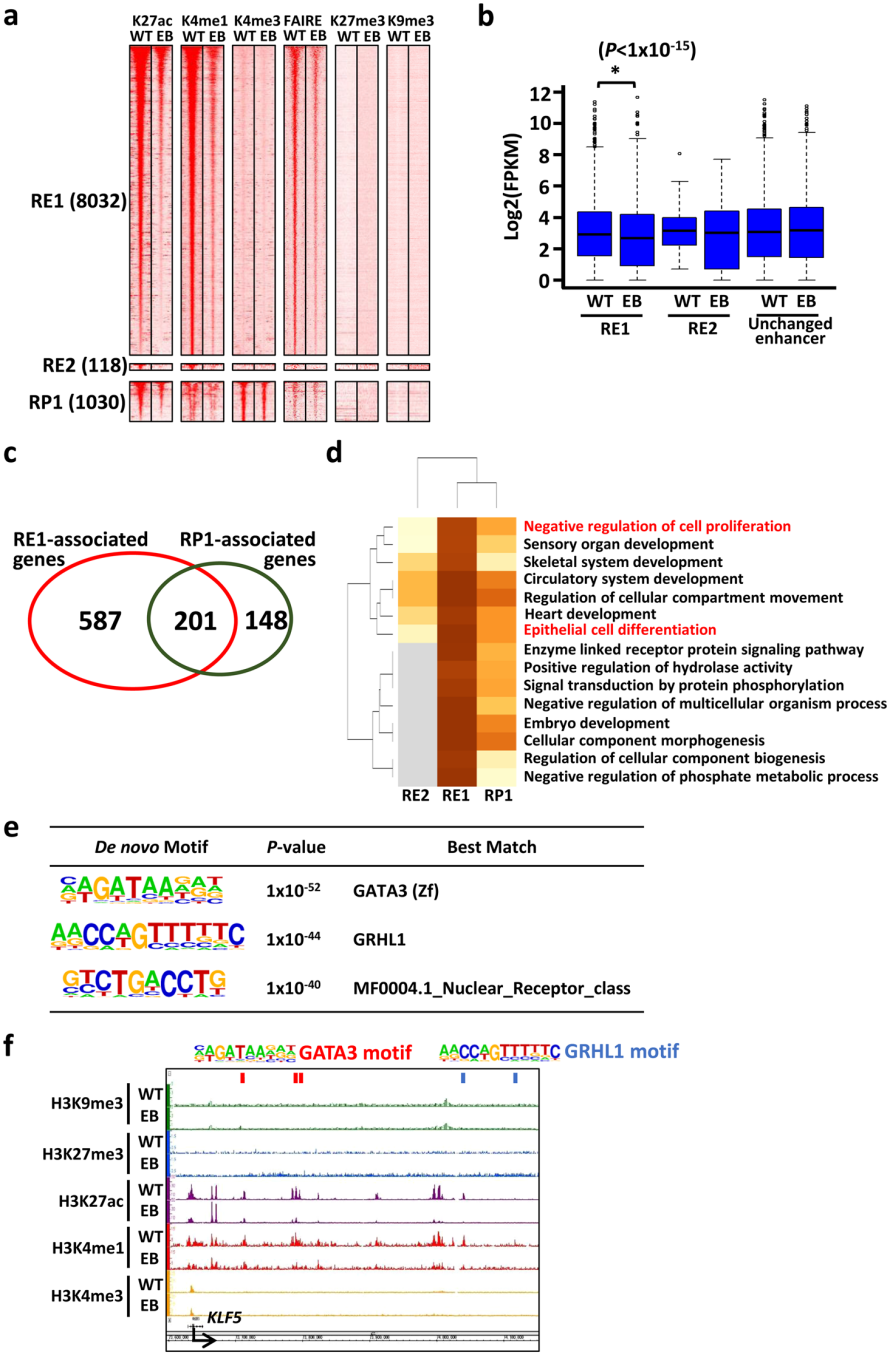
Epigenetic repression of enhancers and promoters in EBV-infected GC cells. *WT*, MKN7 without EBV infection. *EB*, EBV-infected MKN7 cells. (**a**) Heatmaps representing read densities of H3K27ac, H3K4me1, H3K4me3, FAIRE, H3K27me3, and H3K9me3 in ±5-kb regions centred on repressed enhancers and promoters. RE1 and RE2 are subgroups of repressive enhancers in E2 and E7 respectively. RP1 is a subgroup of repressed promoters without repressive mark. (**b**) Expression level of genes nearest to each repressed enhancer and H3K27ac unchanged enhancers. Genes were significantly downregulated in EBV-infected MKN7 cells in RE1 (*P* < 1 × 10^−15^: calculated by paired *t*-test). (**c**) Overlap of RE1-associated genes (*red*) and RP1-associated genes (*green*). This Venn diagram showed that as many as 587 genes were downregulated with enhancer repression without a decrease of H3K27ac at the promoter. (**d**) GO enrichment analysis of genes with enhancer or promoter repression. Colour scale represents binomial *P*-values (in -log_10_ scale). Genes associated with “Negative regulation of cell proliferation” and “Epithelial cell differentiation” were preferentially regulated by both promoter and enhancer repression. (**e**) Enriched known motif at open chromatin regions detected by FAIRE-seq overlapped with RE1. (**f**) Histone modification and position of enriched motifs around the representative repressed gene, *KLF5*.

We next examined repression of enhancers through aberrant deacetylation of H3K27ac in EBV-infected MKN7 cells on genome-wide scale. We defined repression as the reduction of H3K27ac signal to <0.5-fold after EBV infection. We identified 8,032 repressed enhancers without H3K9me3 or H3K27me3 (RE1), 118 repressed enhancers gaining H3K9me3 (RE2), and 1,030 repressed promoters without H3K9me3 or H3K27me3 (RP1) (Fig. 3a). The expression of associated genes of RE1 was significantly reduced after EBV infection (*P* < 1 × 10^−15^; RE1) (Fig. 3b). As many as 587 genes were downregulated by enhancer repression without decrease of H3K27ac at the promoter (Fig. 3c). These results suggest that repression of enhancer regions in EBV-infected MKN7 cells resulted in aberrant gene repression.

To identify repressed targets of RE1 and RP1, GO enrichment analysis was performed. RE1-associated genes showed enrichment of the following categories: positive regulation of differentiation, epithelial cell differentiation, and negative regulation of cell death (Supplementary Table S5). RP1- and RE1-associated genes showed similar enrichment of categories, which suggested that deacetylation at both promoters and enhancers were important to repress gene expression (Supplementary Table S6, Fig. 3d).

To predict transcription factors repressed in EBV-infected MKN7 cells, we performed motif analysis at open chromatin regions detected by FAIRE-seq overlapped with RE1. GATA3 and GRHL1 motifs were enriched at RE1 regions (Fig. 3e). Representative repressed enhancer regions were observed around *KLF5*, which is known as a TSG (Fig. 3f).

### Activation of cancer hallmark genes and repression of TSGs in EBV-infected MKN7 cell line

**Figure 4:**
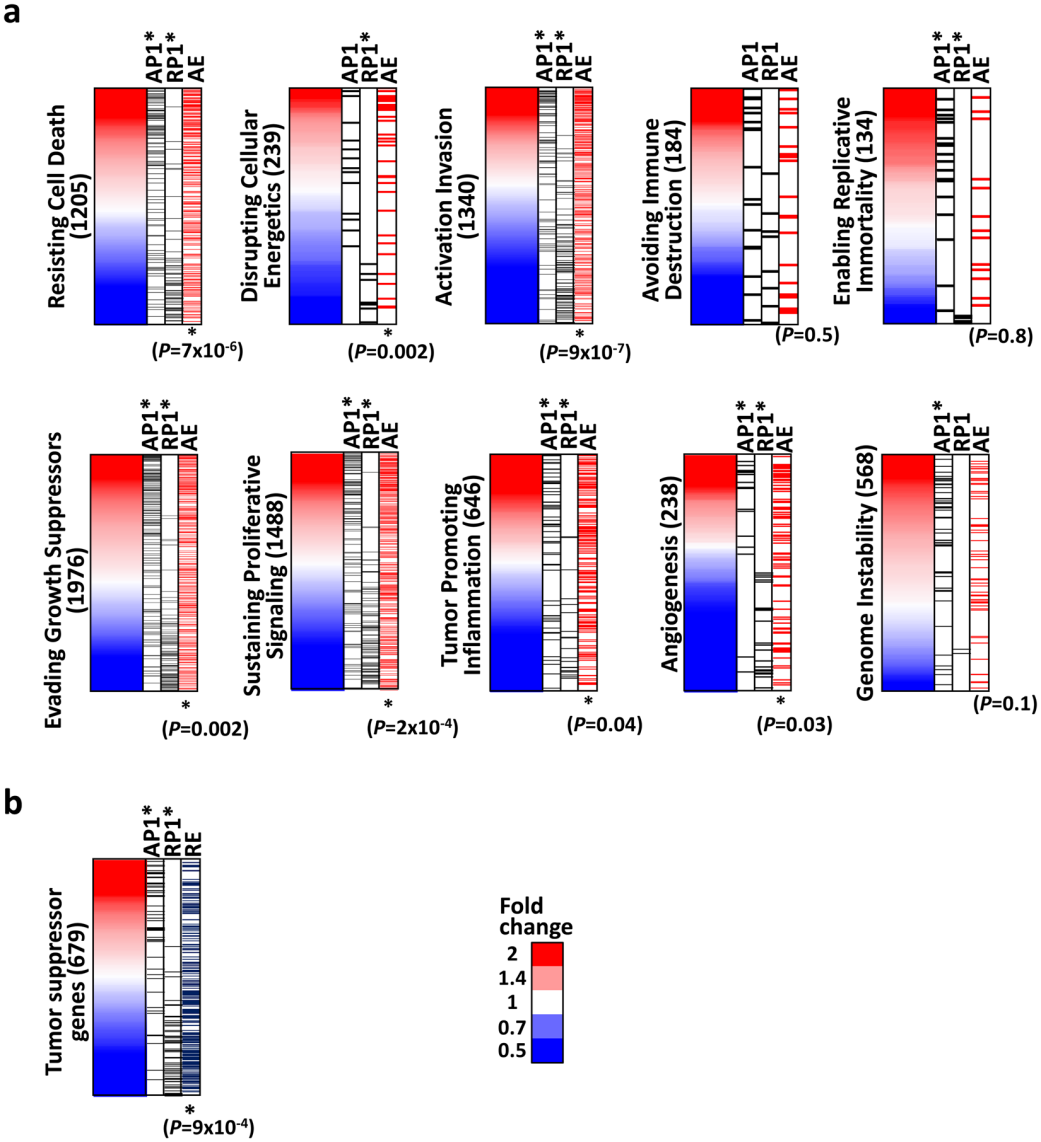
Epigenetic regulation of cancer hallmark genes and tumour suppressor genes. (**a**) Cancer hallmark genes. Genes were sorted according to fold expression changes. *Black bars* show association with activated promoters and repressed promoters, respectively. *Red bars* show association with activated enhancers (AE1). *P*-value was calculated by Kolmogorov-Smirnov test. Enhancer activation was significantly associated with upregulation of cancer hallmark genes, e.g., “Resisting cell death”, “Disrupting cellular energetics”, “Activation invasion”, “Evading growth suppressors”, “Sustaining proliferative signalling”, and “Tumour promoting inflammation”. (**b**) *Black bars* show association with activated promoters and repressed promoters, respectively. *Blue bars* show association with repressed enhancers (RE1). Enhancer repression was significantly associated with repression of tumour suppressor genes.

Aberrant enhancer activation is associated with key oncogenes in several cancers (15, 16). Hanahan and Weinberg proposed that cancer cells acquire a number of hallmark biological capabilities during the multistep process of tumour pathogenesis^31^. In EBV-infected MKN7 cells, 8,319 genes were activated (Supplementary Table S7). Upregulated genes around enhancers activated in EBV-infected MKN7 cells were associated with positive regulation of cell proliferation, differentiation, and cell adhesion (Fig. 2d), and these pathways were included in the hallmarks of cancer^31^. In addition, motif analysis revealed that RUNX1 and ETS1 were related to aberrant enhancer activation and these transcription factors were reported as oncogenes^32–35^. We therefore analysed cancer hallmark genes to elucidate the contribution of activated enhancers to activation of cancer hallmark genes. Of ten cancer hallmarks, EBV-induced active enhancers were significantly associated with activation of genes involved in the following cancer hallmarks: resisting cell death (*P* = 7 × 10^−6^, Kolmogorov-Smirnov test), disrupting cellular energetics (*P* = 0.002, Kolmogorov-Smirnov test), activation invasion (*P* = 9 × 10^−7^, Kolmogorov-Smirnov test), evading growth suppressors (*P* = 0.002, Kolmogorov-Smirnov test), sustaining proliferative signalling (*P* = 2 × 10^−4^, Kolmogorov-Smirnov test), angiogenesis (*P* = 0.03, Kolmogorov-Smirnov test), and tumour promoting inflammation (*P* = 0.04, Kolmogorov-Smirnov test) (Fig. 4a). Thus, enhancers activated in EBV-infected MKN7 cells can stimulate such oncogenic pathways, which might involve traits associated with aggressive GC.

We next analysed possible contribution of repressed enhancers to tumorigenesis. TSGs are inactivated in cancer cells by genetic alteration and/or aberrant DNA methylation at promoter regions resulting in TSG repression. We previously showed that extensive DNA hypermethylation induced by EBV infection results in TSG repression^5^. In EBV-infected MKN7 cells, 8,072 genes were downregulated (Supplementary Table S8). While Ryan *et al*. showed that 17 genes in cancer-related signal transduction pathway are downregulated in EBV-infected AGS cells^28^, eight of them, *BATF*, *COX2*, *TFF1*, *ID2*, *CDK2*, *CCND1*, *CDKN2A*, and *ID4*, were also downregulated in EBV-infected MKN7 cells. Downregulated genes around repressed enhancers showed enrichment of the following categories: negative regulation of cell proliferation and protein phosphorylation, which were associated with TSG functions. Using the TSG database^36, 37^, we determined the association between change in TSG expression and enhancer repression and found that repressed enhancers were significantly associated with TSG repression (*P* = 9 × 10^−4^, Kolmogorov-Smirnov test) (Fig. 4b). Thus, aberrant enhancer regulation by EBV may contribute to tumorigenesis by activating cancer hallmark genes and by repressing TSGs.

### Expression change of cancer-related genes in GC tissue samples

**Figure 5:**
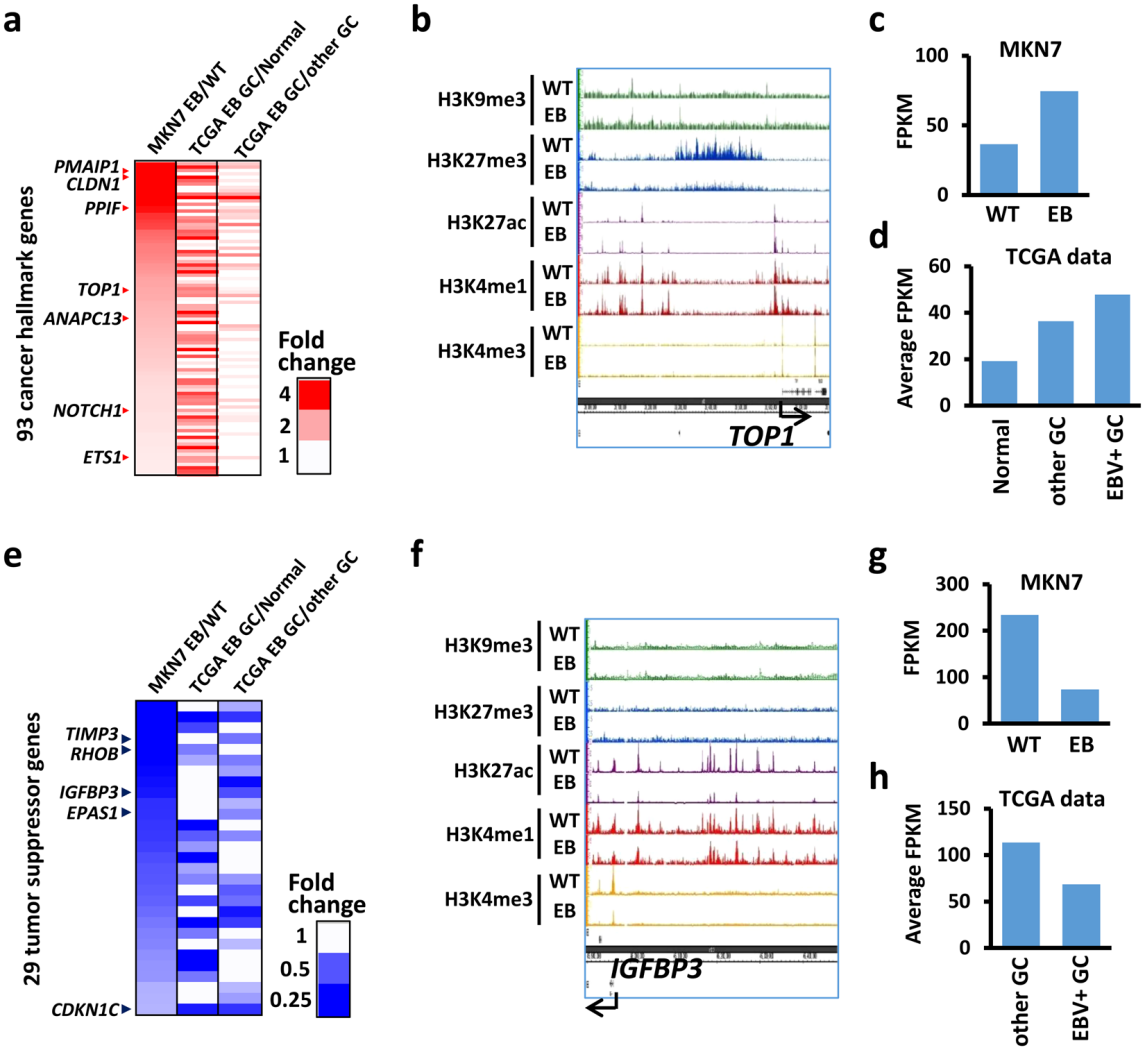
Differentially expressed genes in tissue samples of EBV(+) gastric cancer (GC). *WT*, MKN7 without EBV infection. *EB*, EBV-infected MKN7 cells. (**a**) Fold expression change of cancer hallmark genes in EBV-infected MKN7 cells and EBV(+) GC tissue samples. Among cancer hallmark genes in “Resisting cell death”, “Disrupting cellular energetics”, “Activation invasion”, “Evading growth suppressors”, “Sustaining proliferative signalling”, and “Tumour promoting inflammation”, 92 genes were upregulated in EBV-infected MKN7 cells and in EBV(+) GC tissue samples when compared with normal tissue and/or EBV(−) GC tissue samples. (**b**) Histone modification alteration around a representative cancer hallmark gene in “negative regulation of cell death”, *TOP1*. (**c**) Expression of *TOP1* in WT and EBV-infected MKN7 cells. (**d**) Average expression of *TOP1* in normal gastric tissue, EBV(−) GC, and EBV(+) GC tissue samples. (**e**) Heatmaps representing expression fold change of tumour suppressor genes in EBV cells and EBV(+) GC tissue samples. (**f**) ChIP-seq signal around a representative tumour suppressor gene, *IGFBP3*. (**g**) Expression of *IGFBP3* in WT and EBV-infected MKN7 cells. (**h**) Average expression of *IGFBP3* in EBV(−) GC and EBV(+) GC tissue samples.

We next compared activated cancer-related genes and repressed TSGs in EBV-infected MKN7 cells with differentially expressed genes in EBV(+) GC tissue samples analysed by TCGA^6^, to confirm whether aberrant gene regulation observed in EBV-infected MKN7 cells is also observed in patients with GC (Fig. 5, Supplementary Figs S6, S7). TCGA comprehensively evaluated 295 primary gastric adenocarcinoma, and reported genes differentially expressed in EBV(+) GC tissue samples from normal tissue and/or EBV(−) GC tissue samples. Among genes in the cancer hallmarks “Resisting cell death”, “Disrupting cellular energetics”, “Activation invasion”, “Evading growth suppressors”, “Sustaining proliferative signalling”, “Angiogenesis”, and “ Tumour promoting inflammation”, 93 genes were upregulated in EBV-infected MKN7 cells and in clinical EBV(+) GC tissue samples when compared with normal tissue and/or EBV(−) GC tissue samples (Fig. 5a, Supplementary Table S9). For example, *TOP1* showed enhancer activation and was upregulated in EBV-infected MKN7 cells (Fig. 5b,c), and highly expressed in EBV(+) GCs compared to normal tissue or EBV(−) GC tissue samples (Fig. 5d).

Next, we extracted TSGs whose expression was decreased in EBV(+) GC tissue samples. Twenty-nine TSGs were downregulated in EBV-infected MKN7 cells and in EBV(+) GC tissue samples when compared with normal tissue and/or EBV(−) GC tissue samples (Fig. 5e, Supplementary Table S10). A representative example of such TSG is *IGFBP3*, which is related to the apoptotic process and negative regulation of the cell cycle. A super enhancer observed around *IGFBP3* was repressed in EBV-infected MKN7 cells (Fig. 5f), which resulted in the downregulation of gene expression (Fig. 5g). *IGFBP3* expression was decreased in EBV(+) GC tissue samples when compared to that in EBV(−) GC tissue samples (Fig. 5h). Our results indicate that aberrant activation and repression observed in EBV-infected GC cells is also observed in clinical samples.

## Discussion

We previously showed that aberrant promoter methylation was observed in EBV(+) GCs, and that *in vitro* EBV infection introduced *de novo* DNA methylation in gastric epithelial cells resembling the hypermethylation epigenotype of EBV(+) GC tissue samples^5^. TET family might function as a methylation resistance factor, and its downregulation due to EBV infection might be a cause of genome-wide DNA methylation^12^. Epigenetic alteration induced by EBV infection into gastric epithelial cells is largely unknown, other than DNA methylation at promoter regions^5, 12^. Our results provide evidence that *in vitro* EBV infection in gastric cells induce alteration of global histone modification, including repressive histone marks. Loss of repressive marks and concomitant enhancer activation through increase of H3K4me1 and H3K27ac were observed. Such enhancer activation might contribute to the oncogenic process by activating neighbouring genes. We also showed that enhancer repression through H3K27ac deacetylation and *de novo* DNA methylation might contribute to tumorigenesis by repressing neighbouring TSGs.

We first revealed that differences between promoter regions and enhancer regions overlapped with repressive heterochromatin regions. Consistent with a previous analysis at promoter regions, enhancers losing H3K27ac acquired *de novo* DNA methylation and enhancers gaining H3K27ac maintained low level of DNA methylation. Although promoters with decreased H3K27me3 acquired *de novo* DNA methylation and maintained an inactive state, enhancers losing H3K27me3 did not acquire *de novo* DNA methylation and were activated through increase of H3K27ac. Enhancers losing H3K9me3 were also activated without *de novo* DNA methylation acquisition. Interestingly, promoters losing H3K9me3 did not acquire *de novo* DNA methylation and were activated by H3K27 acetylation. Similar alteration in repressive marks was observed in latent III infection of EBV in LCLs, which was established by EBV infection to resting B cells^38^. Heterochromatin-associated mark impedes the reprogramming of cell identity^39^, and loss of heterochromatin causes cancer^8, 40^. These suggest that EBV infection could induce heterochromatin alteration in both B lymphocytes and gastric epithelial cells to activate genes, which are to be repressed during differentiation, and thus might promote oncogenesis.

Transcription factors activated in EBV-infected GC cells overlapped between gastric epithelial cells and LCLs, resulting in activation of similar enhancer regions. LCL-specific super enhancers such as *MAX* and *ETS1* regions were also activated in EBV-infected MKN7 cells. In LCLs, such super enhancer activation was related to cell growth pathways and these EBV-specific super enhancers were activated by *EBNA2*, *EBNA3A*, *EBNA3C*, and host *NF-κB* ^20^. However, in latent infection with EBV in gastric epithelial cells, only *EBNA1*, *EBER1*, *EBER2*, *LMP2A*, and *BARF0* are expressed, while other regions are inactivated^41^. Our epigenetic data of EBV genome in EBV-infected MKN7 cells were in good agreement with latent state of EBV in gastric epithelial cells. FAIRE-seq data revealed that FR repeats at the OriP were open in EBV-infected MKN7 cells, whereas it was reported that EBNA1 binds to FR repeat region and activate neighbouring genes^30^. These active EBV transcripts might play important roles in epigenomic alteration of host genome, as downregulation of Ten-eleven-translocation (TET) proteins by BARF0 or latent membrane protein 2 A (LMP2A)^12^ and upregulation of DNMT1 by LMP2A^42^ were reported to cause DNA methylation induction in host genome.

Meanwhile, expression of host epigenetic factors was also altered in EBV-infected GC cells. In EBV-infected MKN7 cells, *TET2* expression was downregulated as mentioned above (Supplementary Table S8) and *DNMT1* was upregulated (Supplementary Table S7). While these expression alterations might contribute to epigenetic repression of promoters and enhancers, *EP300* was upregulated (Supplementary Table S7) and *HDAC7*, *HDAC8*, *HDAC9*, *HDAC10*, and *HDAC11* were downregulated (Supplementary Table S8) in EBV-infected MKN7 cells. It might be suggested that these expression alterations of HAT and HDACs might contribute to enhancer activation. Expression of *MLL3* and *MLL4*, which are known as lysine methyl transferases at enhancer regions, however, was not changed. Further investigations are necessary to fully clarify which virus or host factors induce host chromatin alteration in gastric epithelial cells.

Enriched motifs, e.g., RUNX1 and ETS1, at activated enhancer regions in EBV-infected MKN7 cells were similar to those in LCLs^20^. Runx1 is a key developmental regulator in hematopoietic stem cells^43^ and often involved in leukaemia^32^. Recent reports showed that *RUNX1* is expressed in epithelial cells of several organs and presents stem cell functions^44–46^. Matsuo *et al*. demonstrated *RUNX1* expression in stem cells of the stomach corpus and antrum^47^. These reports suggest that aberrant *RUNX1* expression in gastric epithelial cells might deregulate the differentiated state and promote cancer. *ETS1* is a proto-oncogene associated with several cancers^33, 34^, including GC^35^. Therefore, host transcription factors activated by EBV infection might regulate EBV-specific enhancers to promote oncogenesis. We showed that enhancer activation might contribute to upregulation of cancer hallmark genes, and altered expression of these genes was confirmed in clinical EBV(+) GC specimens. These upregulated cancer hallmark genes or their activated enhancer regions could be used as drug targets to prevent cancer development.

Saha *et al*. reported TSG silencing by promoter DNA methylation in EBV-infected cells using B lymphocytes^25^. While there is no report about enhancer repression by aberrant DNA methylation in EBV-infected GC cells, we revealed that *de novo* DNA methylation was introduced not only to promoter regions, but also to active enhancer regions, and the increase of DNA methylation together with loss of active histone marks at enhancer regions might contribute to TSG repression.

We showed that the GATA3 and GRHL1 motifs were enriched at repressed enhancers. *GATA3* is a tumour suppressor, and decreased expression of *GATA3* is associated with poor prognosis in primary gastric adenocarcinoma^48^. *GRHL1* is also a tumour suppressor in neuroblastoma^49^. *GRHL2*, whose binding motif is similar to that of *GRHL1*, functioned as a tumour suppressor, and its expression reduces invasion and migration in GC^50^. Their expression was not changed in EBV-infected MKN7 cells, but repression of their binding enhancers might promote oncogenesis by repressing their downstream pathways.

Although our comprehensive analysis suggested that enhancer alteration could contribute to carcinogenesis, and may provide a novel target to facilitate therapeutic strategy, genome editing or epigenome editing at predicted enhancers are necessary to fully clarify contribution of each enhancer to transcription regulation. Recently, CRISPR/Cas9 based technologies have been developed and enable us to perform site-specific editing of genome/epigenome^51, 52^. These analyses would elucidate targets of each enhancer and transcription regulation by enhancers, and be helpful for development of therapeutic strategy.

In summary, dynamic epigenetic activation and repression occur in enhancer regions in EBV-infected GC cells, including gain/loss of H3K27ac, loss of H3K27me3, relocation of H3K9me3, and acquisition of *de novo* DNA methylation. These epigenetic alterations at enhancer regions are markedly associated with imbalance of oncogenes and TSGs.

## Methods

### Cell culture

The human GC cell line, MKN7, which exhibits low DNA methylation epigenotype^5^, was purchased at Riken BioResource Center Cell Bank (Ibaraki, Japan) and cultured in RPMI-1640 medium supplemented with 10% FBS and penicillin/streptomycin. MKN7 cells were infected with EBV-Akata recombinant virus carrying a neomycin resistance gene^53^ and EBV-infected MKN7 cells were selected by G418 (Roche Diagnostics, Basel, Switzerland) at 200 μg/mL as previously reported^5^. The study design was approved by the institutional review board in Chiba University and The University of Tokyo, and the study was carried out in accordance with the institutional guidelines.

### Chromatin Immunoprecipitation (ChIP) and library construction

ChIP assays were performed from approximately 10^7^ cells as previously reported^54^. Cells were crosslinked with 1% formaldehyde for 10 min at room temperature and formaldehyde was quenched by addition of 2.5 M glycine to a final concentration of 0.125 M. Crosslinked chromatin was sonicated to a size of 0.2–1 kb using an ultrasonic disruptor, BRANSON Digital Sonifier (BRANSON, Danbury, CT, USA ). A total of 2–5 μg of antibody and 20 μL of Protein G sepharose beads or 20 μL of anti-Mouse IgG Dynal magnetic beads were mixed in IP dilution buffer and incubated for 6 hour at 4 °C. After washing with IP dilution buffer, antibody-binding beads were added to the sonicated-chromatin sample and incubated overnight at 4 °C. Beads were washed and chromatin was eluted, followed by reversal of the crosslinking and DNA purification. Chromatin-immunoprecipitated DNA was dissolved in EB buffer (Qiagen, Hilden, Germany). Libraries were constructed by using NEBNext ChIP-seq Library Prep Reagent Set for Illumina (NEB, Ipswich, MA, USA) according to the manufacturer’s instructions. ChIP-seq libraries were quantified by Bioanalyzer (Agilent, Santa Clara, CA, USA) and sequenced at a concentration of 4 pM on an Illumina Hiseq (Illumina, San Diego, CA, USA).

### FAIRE-seq

FAIRE experiment were performed as previously reported^55^. Cells were crosslinked with 1% formaldehyde for 10 min at room temperature and formaldehyde was quenched by addition of 2.5 M glycine to a final concentration of 0.125 M. Crosslinked chromatin was sonicated to a size of 0.2–1 kb using an ultrasonic disruptor (BRANSON Digital Sonifier). Aliquot was taken, de-crosslinked, purified by phenol/chloroform extraction, and run on a gel to ensure average fragment sizes of 300 bp. Remaining samples were processed three times by phenol/chloroform extraction to recover DNA not bound by nucleosome in the water phase. The samples were de-crosslinked by overnight incubation at 65 °C and purified by ethanol precipitation. They were subsequently treated with RNase A (final 50 ug/ml), purified by QIAquick PCR purification kit (Qiagen). Libraries were constructed by using NEBNext ChIP-seq Library Prep Reagent Set for Illumina (NEB) according to the manufacturer’s instructions. The ChIP DNA was used to prepare library samples following the manufacturer’s instructions (Illumina). Deep sequencing was performed on the Solexa Genome Analyzer II to obtain 36-bp single reads.

### ChIP sequencing (ChIP-seq) and FAIRE-seq analysis

Sequenced reads in ChIP-seq experiment were mapped to UCSC human genome (hg19) using bowtie. Duplicated reads were removed with Picard tools. Peak calling and motif analysis were performed by using HOMER software (http://homer.salk.edu/homer/index.html)^56^. HOMER was also used to get differential peaks. Enhancer annotation to the nearest genes was performed by using GREAT (http://bejerano.stanford.edu/great/public/html/index.php)^57^. Peak heatmaps were produced with the use of HOMER and TreeView for enrichment calculation and visualisation. H3K27me3 distribution at genetic regions was produced using NGSplot^58^. To divide genomic regions based on histone modification status, ChromHMM analysis was performed^59^. Gene Ontology (GO) analysis was performed by using Metascape (http://metascape.org/gp/index.html#/main/step1)^60^.

For analysis on histone modification of EBV genome, sequenced reads in ChIP-seq experiment were mapped to Akata-EBV genome^61^.

### Library construction for RNA sequencing (RNA-seq)

RNA was extracted by using the RNeasy Mini Kit (Qiagen) following the manufacturer’s protocol, and treated with DNaseI (Qiagen). Libraries for RNA-seq were prepared using the TruSeq Stranded mRNA Sample Prep Kit (Illumina), following the manufacturer’s protocol. Deep sequencing was performed on the Illumina HiSeq. 1500 or NextSeq. 500 platform using the TruSeq Rapid SBS Kit (Illumina) in 50-base single-end mode according to the manufacturer’s protocol.

### RNA-seq analysis

Sequenced reads from RNA-seq experiment were aligned by using TopHat, and Cufflinks was used for transcript assembly. Gene expression levels were expressed as fragments per kilobase of exon per million mapped sequence reads (FPKM).

### Infinium assay

Genome-wide DNA methylation analysis was performed by using Infinium HumanMethylation450 BeadChip (Illumina) as previously described^12^. Briefly, bisulphite conversion was performed by using the Zymo EZ DNA Methylation Kit (Zymo Research, Irvine, CA, USA) with 500 ng of genomic DNA for each sample. Whole genome amplification, labelling, hybridisation, and scanning were performed according to the manufacturer’s protocols. Overlapping probes of histone modification changed regions were extracted with Bedtools. The Infinium HumanMethylation450 BeadChip (Illumina) contains approximately 485,000 individual CpG sites, covering 99% of RefSeq genes with an average of 17 CpG sites per gene. In each CpG site, the ratio of the fluorescent signal, so-called β value, was measured by a methylated probe relative to the sum of both methylated and unmethylated probe. The β values range from 0.00 to 1.00 and reflect the methylation level of each CpG site, from low to high.

### Cancer Hallmark analysis

Genes included in the following GO categories were used for cancer hallmark analysis. GO:0001525 was for Angiogenesis. GO:0032200, GO:0090398, and GO:0090399 were for Enabling Replicative Immortality. GO:0034330, GO:0016477, GO:0010718, and GO:0007155 were for Activating Invasion. GO:0006281, GO:0051383, GO:0007065, GO:0000819, GO:0051988, GO:0030997, GO:0046605, GO:0090224, GO:0010695, and GO:0031577 were for Genome Instability. GO:0060548, GO:0012501, and GO:0010941 were for Resisting Cell Death. GO:0006091 was for Disrupting Cellular Energetics. GO:0007166 and GO:0070848 were for Sustaining Proliferative Signalling. GO:0006954 and GO:0045321 were for Tumour-Promoting Inflammation. GO:0002507, GO:0001910, GO:0019882, and GO:0002767 were for Avoiding Immune Destruction. GO:0007049 and GO:0008283 were for Evading Growth Suppressors (Supplementary Table S11).

### TSG analysis

We obtained the list of TSG from TUMOR SUPPRESSOR GENE DATABASE (https://bioinfo.uth.edu/TSGene/index.html)^36, 37^.

### Statistical analysis

All statistical analyses were performed using R-2.15.3. The *P*-values for DNA methylation and expression analysis were obtained using paired *t*-test to evaluate differences between two groups, with *P* < 0.05 considered statistically significant. The *P*-values for cancer hallmark analysis and TSG analysis were obtained using Kolmogorov-Smirnov test to evaluate difference in distribution against uniform distribution, with *P* < 0.05 considered statistically significant.

### Data availability

Infinium data were submitted to the GEO DataSets, and the accession number is GSE89269. RNA-seq data data were submitted to the GEO DataSets, and the accession number is GSM2253673 - GSM2253676. H3K4me3, H3K27ac, and H3K27me3 ChIP-seq data data were submitted to the GEO DataSets, and the accession number is GSE97837. H3K4me1 and H3K9me3 ChIP-seq data and FAIRE-seq data were submitted to the GEO DataSets, and the accession number is GSE97838.

## Electronic supplementary material

Supplementary Figures

Supplementary Dataset 1
