# Supplementary material for: Regulation of tumour related genes by dynamic epigenetic alteration at enhancer regions in gastric epithelial cells infected by Epstein-Barr virus
Source: Sci Rep. 2017 Aug 11;7:7924. doi: 10.1038/s41598-017-08370-7 (PMC5554293; doi:10.1038/s41598-017-08370-7)
Supplement: Supplementary file 1 — Supplementary Figures [file 41598_2017_8370_MOESM1_ESM.pdf]

Supplementary Figures

Regulation of tumour related genes by dynamic epigenetic alteration at enhancer regions in gastric epithelial cells infected by Epstein-Barr virus

Atsushi Okabe, Sayaka Funata, Keisuke Matsusaka, Hiroe Namba, Masaki Fukuyo, Bahityar Rahmutulla, Motohiko Oshima, Atsushi Iwama, Masashi Fukayama, Atsushi Kaneda

Supplementary Figures:

Supplementary Figure S1

Supplementary Figure S2

Supplementary Figure S3

Supplementary Figure S4

Supplementary Figure S5

Supplementary Figure S6

Supplementary Figure S7

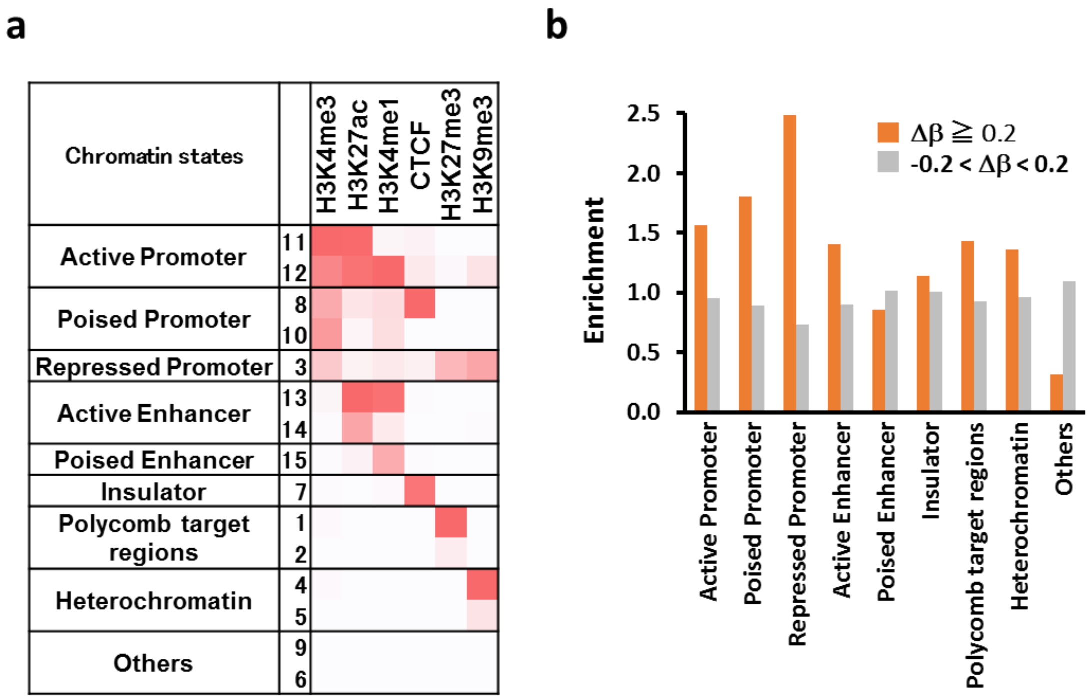

Supplementary Figure S1. Chromatin state and *de novo* DNA methylation. (a) Heatmaps of histone modification enrichment at each chromatin states in MKN7 cells without EBV infection (WT). Genomic regions were divided by ChromHMM analysis. The genomic region was divided into 9 different chromatin states in WT cells. (b) Ratios of the number of probes with *de novo* DNA methylation induced by EBV infection in MKN7 cells ($\Delta\beta \geqq 0.2$) and the number of unmethylated probes ($-0.2 \leqq \Delta\beta < 0.2$) in each chromatin state against expected number of probes in each chromatin state. *De novo* DNA methylation induction by EBV infection was preferentially observed at repressive and poised promoter regions. In addition, active enhancer, polycomb target regions, and heterochromatin regions also acquired DNA methylation.

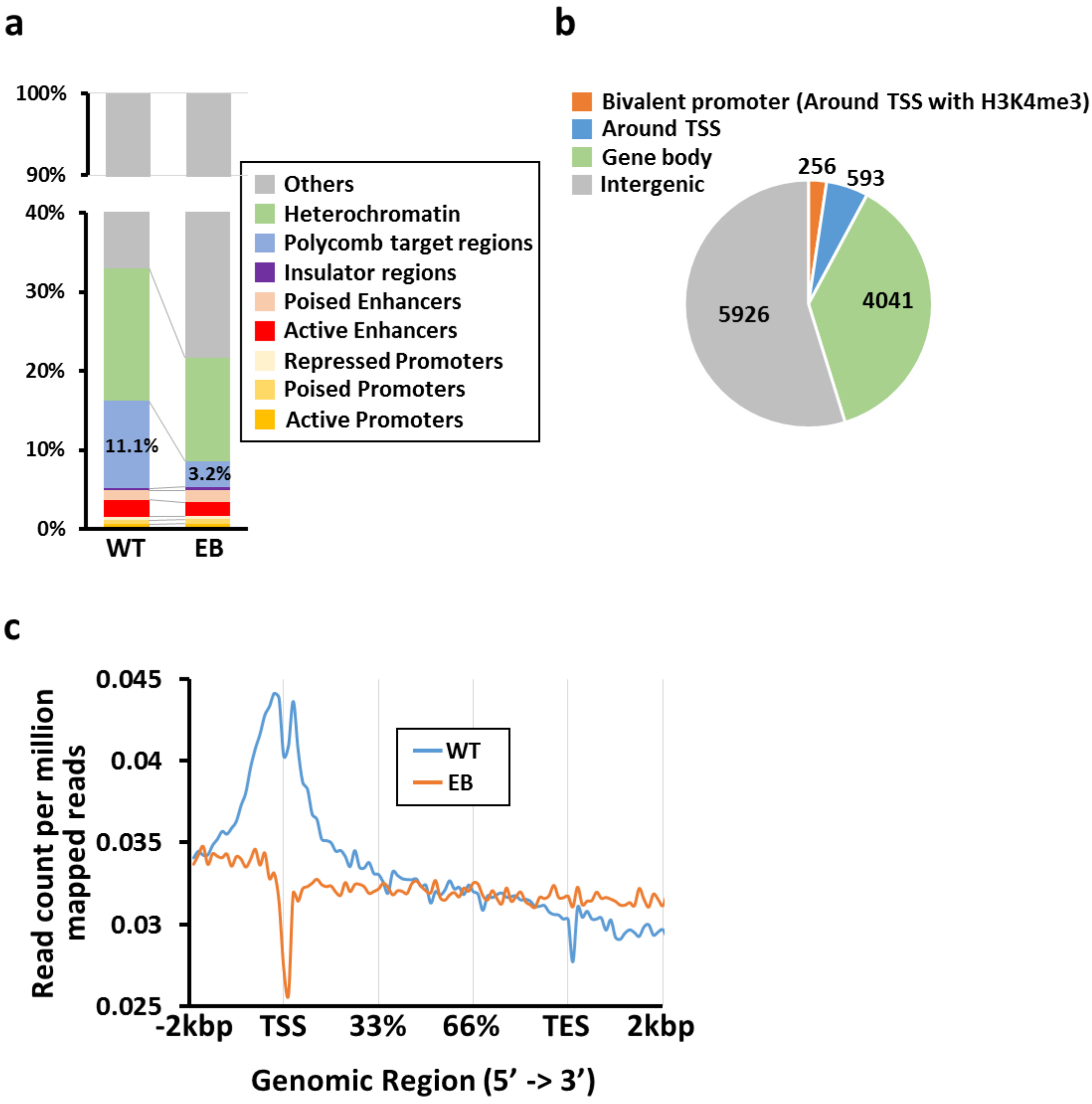

Supplementary Figure S2. H3K27me3 loss in EBV-infected GC cells. TSS, transcription start site. TES, transcription end site. *WT*, MKN7 without EBV infection. *EB*, EBV-infected MKN7 cells. (a) The relative percentage of the genome represented by each chromatin state in WT and EBV-infected MKN7 cells. Polycomb target regions in WT cells occupied 11.1% of the genomic region and it was reduced to 3.2% in EBV-infected cells. (b) The proportion of annotated regions which lost H3K27me3. Most of H3K27ac reduced regions were overlapped with gene body or intergenic regions. (c) ChIP-seq read counts of H3K27me3 and relative distance to TSS and TES. H3K27me3 level was significantly reduced around TSS in EBV-infected MKN7 cells.

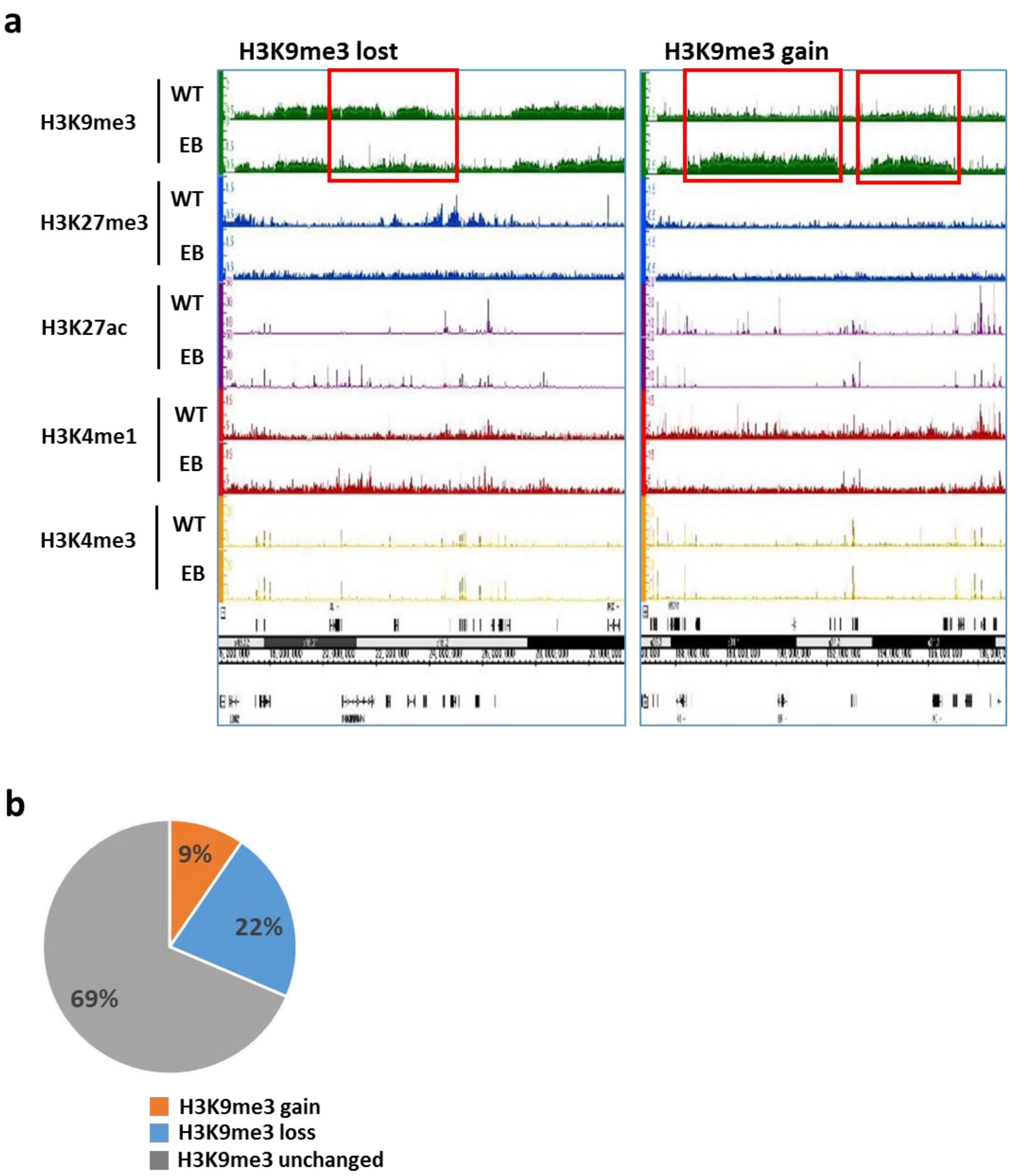

Supplementary Figure S3. H3K9me3 relocation in EBV-infected MKN7 cells. *WT*, MKN7 without EBV infection. *EB*, EBV-infected MKN7 cells. (a) Inverse correlation of H3K9me3 and active marks. H3K9me3 change was inversely correlated with active marks, H3K4me1, and H3K27ac. Regions of H3K9me3 loss with gain of active marks, and H3K9me3 gain with loss of active marks, were representatively shown. (b) The proportion of changes in H3K9me3 peaks. 9% of H3K9me3 enriched regions showed gain of H3K9me3 level and 22% of those showed decrease of H3K9me3 level.

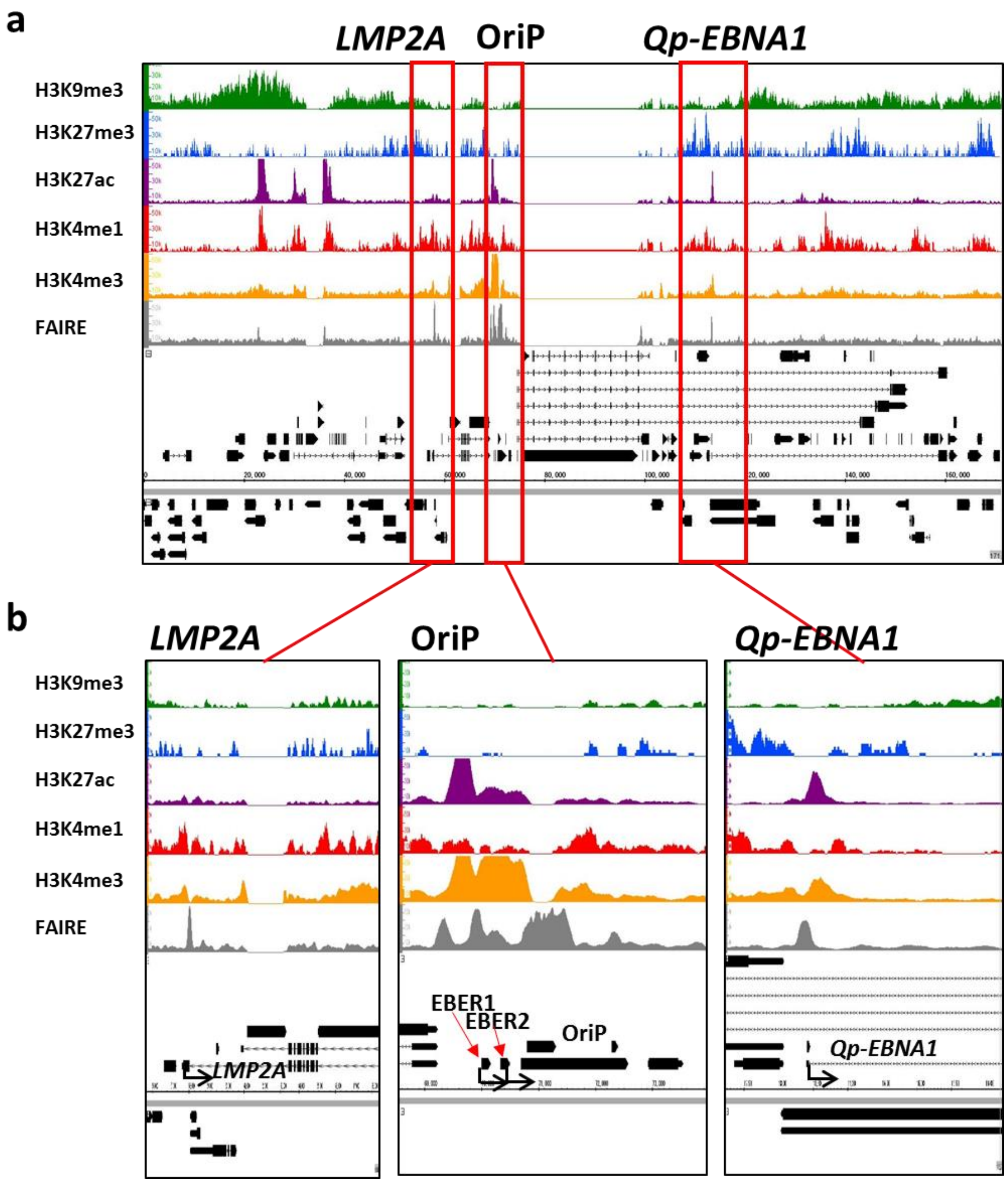

Supplementary Figure S4. Epigenetic status of EBV genome in EBV-infected MKN7 cells. (a) Whole image of ChIP-seq and FAIRE-seq signals of EBV genome. Most region of EBV genome was modified with repressive marks, e.g. H3K27me3 and H3K9me3. (b) Epigenetic status around LMP2A, OriP, and Qp-EBNA1. Qp-EBNA1, which is important to maintain latent state, and OriP were modified with H3K27ac. Strong FAIRE signal was observed at LMP2A region.

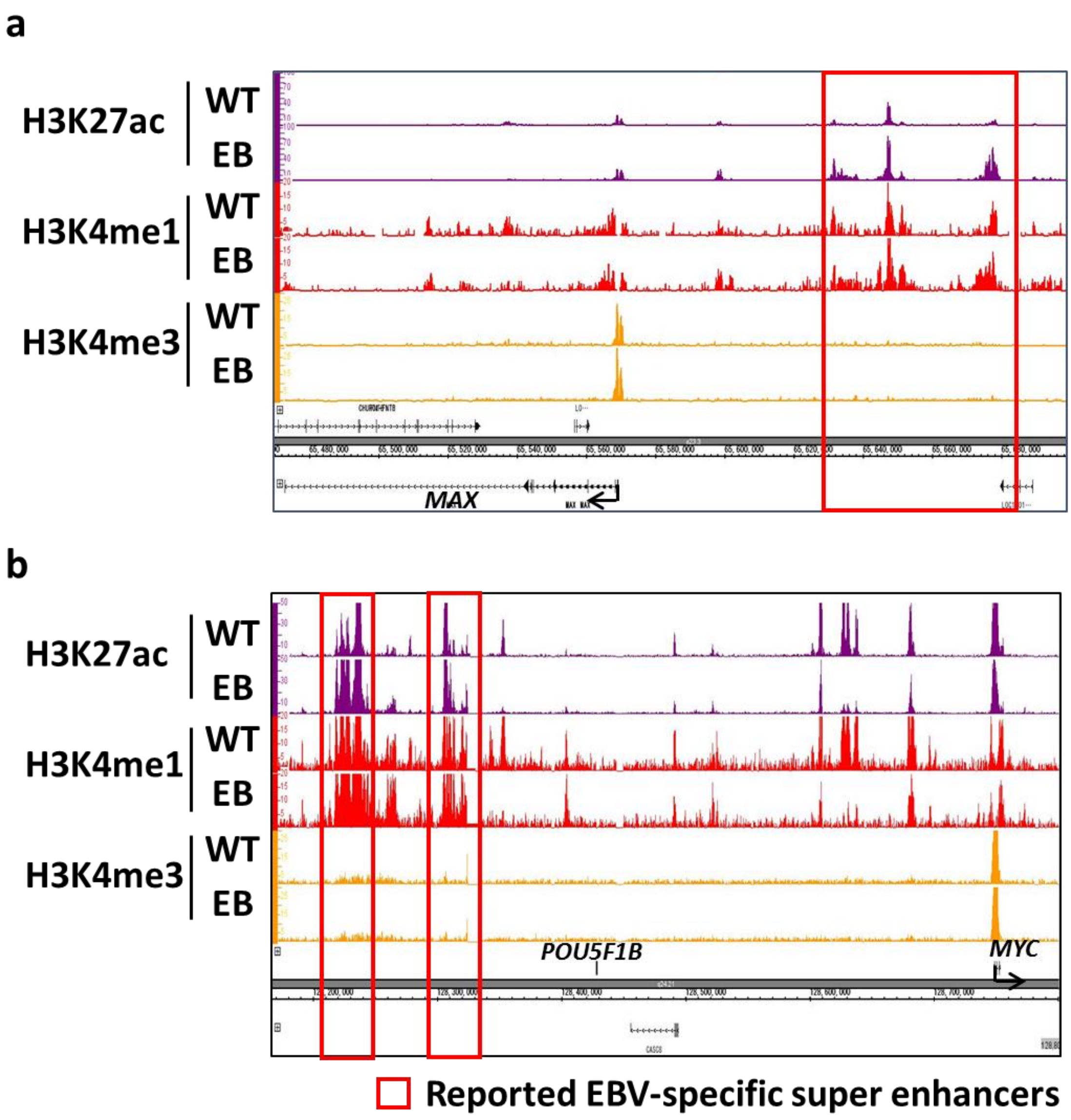

Supplementary Figure S5. Reported enhancer regions targeted by EBV infection. *WT*, MKN7 without EBV infection. *EB*, EBV-infected MKN7 cells. (a) ChIP-seq signal around the reported EBV target gene, *MAX*. Enhancers around *MAX* gene were activated in EBV-infected MKN7 cells. (b) ChIP-seq signal around the reported EBV target gene, *MYC*. *MYC* enhancers were at active state in EBV-infected MKN7 cells though they were already activated before EBV infection.

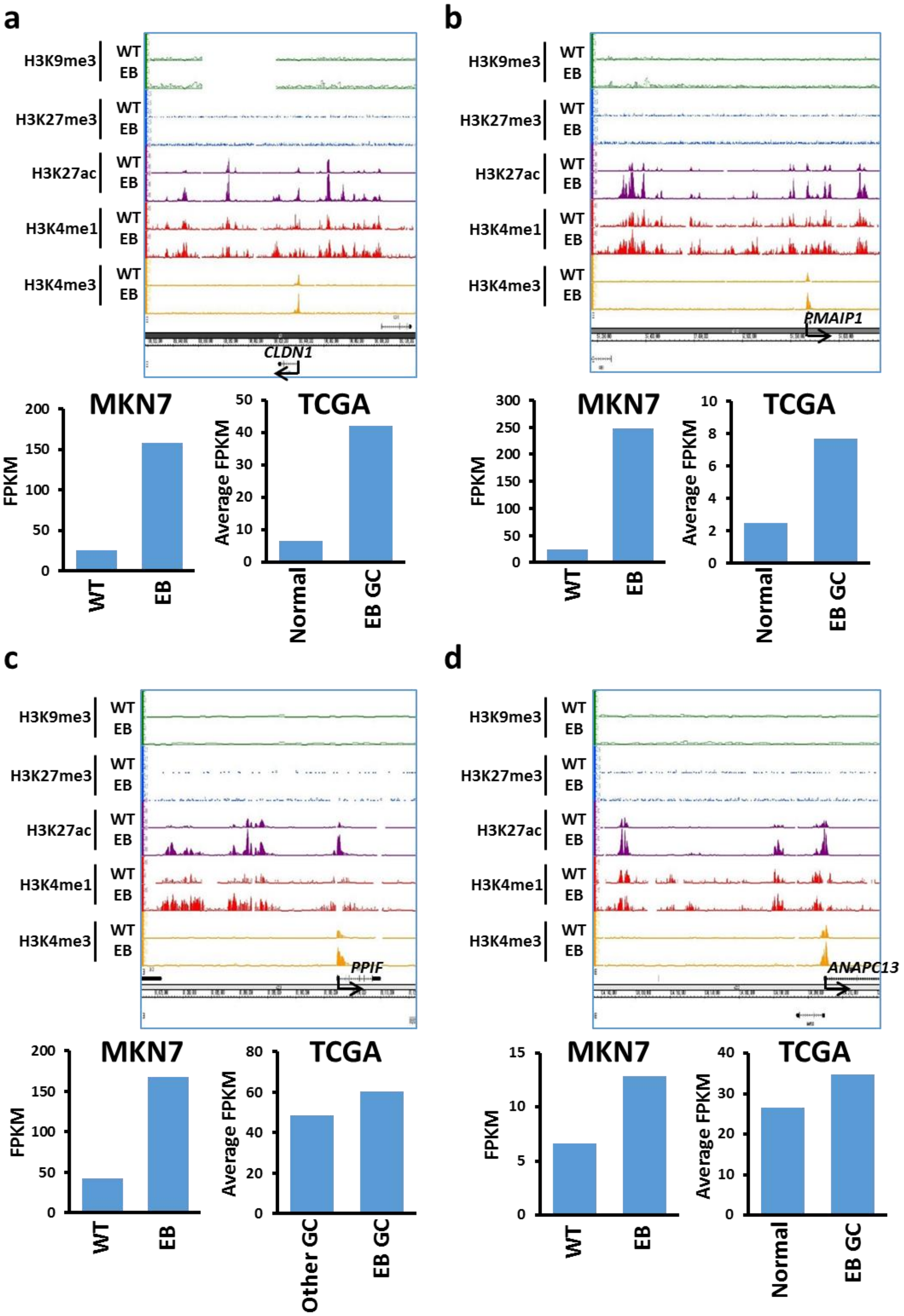

Supplementary Figure S6. Representative cancer hallmark genes upregulated in

TCGA EBV(+) GC tissue samples. *WT,* MKN7 without EBV infection. *EB,* EBV-infected MKN7 cells. (a) ChIP-seq signal around a representative gene of cancer hallmarks termed as “Activation invasion”, *CLDN1* (*upper*). Expression of *CLDN1* in WT and EBV-infected MKN7 cells and the average of *CLDN1* expression levels in normal gastric tissue and EBV(+) GC tissue samples (*bottom*). (b) ChIP-seq signal around a representative gene of cancer hallmarks termed as “Sustaining Proliferative signalling”, *PMAIP1* (*upper*). Expression of *PMAIP1* in WT and EBV-infected MKN7 cells and the average of *PMAIP1* expression levels in normal gastric tissue and EBV(+) GC tissue samples (*bottom*). (c) ChIP-seq signal around a representative gene of cancer hallmarks termed as “Disrupting cellular energetics”, *PPIF* (*upper*). Expression of *PPIF* in WT and EBV-infected MKN7 cells and the average of *PPIF* expression levels in EBV(−) GC and EBV(+) GC tissue samples (*bottom*). (d) ChIP-seq signal around a representative gene of cancer hallmarks termed as “Evading growth suppressors”, *ANAPC13* (*upper*). Expression of *ANAPC13* in WT and EBV-infected MKN7 cells and the average of *ANAPC13* expression levels in normal gastric tissue and EBV(+) GC tissue samples (*bottom*).

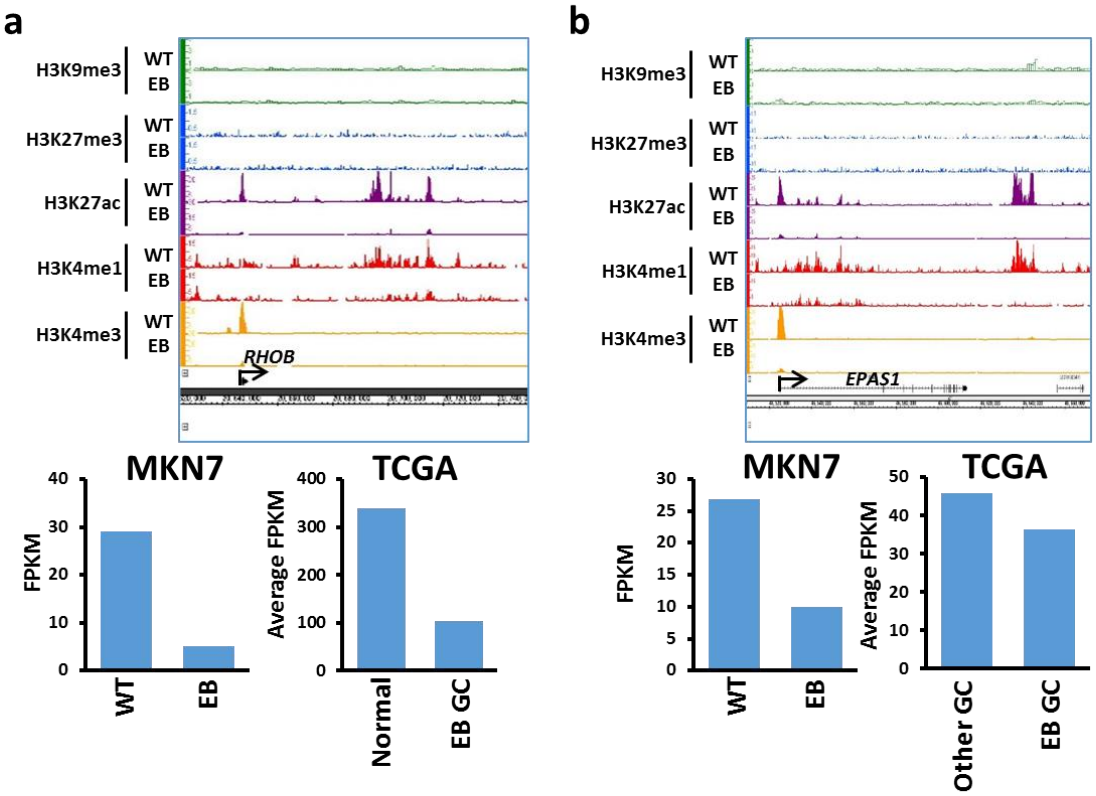

Supplementary Figure S7. Representative tumour suppressor genes downregulated in TCGA EBV(+) GC tissue samples. *WT*, MKN7 without EBV infection. *EB*, EBV-infected MKN7 cells. (a) ChIP-seq signal around a representative tumour suppressor gene, *RHOB* (*upper*). Expression of *RHOB* in WT and EBV-infected MKN7 cells and the average of *RHOB* expression levels in normal gastric tissue and EBV(+) GC tissue samples (*bottom*). Downstream enhancer regions of *RHOB* were repressed in EBV-infected MKN7 cells and *RHOB* expression was repressed concomitantly. *RHOB* repression was also observed in EBV(+) GCs. (b) ChIP-seq signal around a representative tumour suppressor gene, *EPAS1* (*upper*). Expression of *EPAS1* in WT and EBV-infected MKN7 cells and the average of EPAS1 expression levels in EBV(–) GC and EBV(+) GC tissue samples (*bottom*). Downstream enhancer regions of *EPAS1* were repressed in EBV-infected MKN7 cells and *EPAS1* expression was repressed concomitantly. *EPAS1* repression was also observed in EBV(+) GCs.
